# A nomogram to predict the probability of axillary lymph node metastasis in female patients with breast cancer in China: A nationwide, multicenter, 10-year epidemiological study

**DOI:** 10.18632/oncotarget.13330

**Published:** 2016-11-12

**Authors:** Jian Zhang, Xiao Li, Rong Huang, Wei-Liang Feng, Ya-Nan Kong, Feng Xu, Lin Zhao, Qing-Kun Song, Jing Li, Bao-Ning Zhang, Jin-Hu Fan, You-Lin Qiao, Xiao-Ming Xie, Shan Zheng, Jian-Jun He, Ke Wang

**Affiliations:** ^1^ Department of Breast Surgery, The First Affiliated Hospital of Xi’an Jiaotong University, Xi’an, P.R. China; ^2^ Department of Cancer Epidemiology, Cancer Institute & Hospital, Chinese Academy of Medical Sciences & Peking Union Medical College, Beijing, P.R. China; ^3^ Department of Epidemiology, West China School of Public Health, Sichuan University, Chengdu, Sichuan, P.R. China; ^4^ Department of Breast Surgery, Zhejiang Cancer Hospital, Hangzhou, P.R. China; ^5^ Department of Breast Oncology, Sun Yat-Sen University Cancer Center, Guangzhou, P.R. China; ^6^ Department of Breast-thyroid Surgery, Xiangya Second Hospital, Central South University, Changsha, P.R. China; ^7^ Department of Breast Surgery, Liaoning Cancer Hospital, Shenyang, P.R. China; ^8^ Center of Breast Disease, Cancer Institute & Hospital, Chinese Academy of Medical Sciences & Peking Union Medical College, Beijing, P.R. China; ^9^ Department of Pathology, Cancer Institute & Hospital, Chinese Academy of Medical Sciences & Peking Union Medical College, Beijing, P.R. China

**Keywords:** breast cancer, axillary lymph node metastasis, prediction model, nomogram

## Abstract

Axillary lymph node dissection (ALND) or sentinel lymph node biopsy (SLNB) alone may lead to postoperative complications. Among patients with positive ALN in the preoperative examination, approximately 40% patients do not have SLN metastasis. Herein, we aimed to develop a model to predict the probability of ALN metastasis as a preoperative tool to support clinical decision-making. We retrospectively analyzed the clinicopathological features of 4211 female patients with breast cancer who were diagnosed in seven breast cancer centers representing entire China, over 10 years (1999-2008). The patients were randomly categorized into a training cohort or validation cohort (3:1 ratio). Multivariate logistic regression analysis was performed for 1869 patients with complete information on the study variables. Age at diagnosis, tumor size, tumor quadrant, clinical nodal status, local invasion status, pathological type, and molecular subtypes were the independent predictors of ALN metastasis. The nomogram was then developed using the seven variables. Further, it was subsequently validated in 642 patients with complete data on variables in the validation cohort. Coefficient of determination (R^2^) and the area under the receiver-operating characteristic (ROC) curve (AUC) were calculated to be 0.979 and 0.7007, showing good calibration and discrimination of the model, respectively. The false-negative rates of the nomogram were 0 and 6.9% for the predicted risk cut-off values of 14.03% and 20%, respectively. Therefore, when the predicted risk is less than 20%, SLNB may be avoided. After further validation in various patient populations, this model may support increasingly limited axillary surgery in breast cancer.

## INTRODUCTION

Breast cancer is the most common malignancy in women, accounting for 25% of all female cancer cases and 15% of all cancer-related deaths [1]. Recently, breast cancer incidence has plateaued [2]. The metastasis status of axillary lymph nodes (ALN) is an important factor affecting the prognosis of patients with breast cancer, a major component of breast cancer staging, and an important basis for designing treatment programs [3–5]. The sentinel lymph-node biopsy (SLNB) has been rapidly replacing ALN dissection (ALND) to become the standard surgical procedures for early breast cancer patients with clinical negative axillary lymph nodes [6–8].

Although SLNB is landmark progress in the field of surgery and can avoid unnecessary ALND for patients, a discussion of the disadvantages related to SLNB should not be omitted. Because of the need to assess the pathological state of SLN during surgery, this procedure is time consuming and expensive. Besides, although it causes less damage than ALND [9, 10], SLNB involves a certain degree of side injury, including upper limb edema, shoulder and back pain, arm numbness [11], weakened shoulder, and reduced arm strength [12]. Therefore, with either ALND or SLNB alone, postoperative associated complications may occur. In addition, studies have reported that among the patients with absence of ALN on preoperative clinical examination, more than 60% patients do not have SLN metastasis. Further, even in the patients with ALN on preoperative clinical examination, approximately 40% patients do not have SLN metastasis [13]. Therefore, it is important to screen patients with ALN and identify patients without SLN metastasis before surgery in order to avoid unnecessary SLNB. To this effect, researchers are attempting to determine methods to avoid unnecessary SLNB or ALND.

Medical Centers outside China have published models to predict the ALN status in patients. For example, the Memorial Sloan Kettering Cancer Center (MSKCC) developed a nomogram that was used for preoperative prediction of SLN status and prediction of non-SLN status when SLN was present. This model was verified in many medical centers and widely accepted, as it helped clinicians decide the surgical procedure required for regional lymph nodes before the surgery. However, there were several inconsistencies in the verification results between populations, which could have occurred due to differences in race, social and cultural background, level of economic development, level of medical care, and many other factors [14–19].

Prediction models are often built using clinical and pathological data of a specific population. Therefore, when used to predict disease in another group of people, their predictive value is limited. To our knowledge, the report on the establishment of prediction model of ALN metastasis in China was few at present, and these models included data from single-center studies that were not fully representative of the entire population of China.

Therefore, in this study, we aimed to (1) represent the entire population of China by retrospectively analyzing relevant medical records of female patients with breast cancer who were diagnosed over a period of 10 years, (2) determine the risk factors of ALN metastasis in breast cancer, and (3) build a prediction model of ALN metastasis in breast cancer in order to help clinicians in the decision-making process.

## RESULTS

### Clinicopathologic features and grouping of patients

Of the 4,211 patients, 3158 were included in the training cohort and 1053 were included in the validation cohort in a 3:1 ratio. The clinical and pathological data of the patients between the two groups did not differ significantly (*p* > 0.05), which was consistent with the randomization. Among patients who underwent SLNB and ALND, 48.74% (1426/2926) had ALN metastasis in the training cohort and 49.59% (483/973) had ALN metastasis in the validation cohort (Table 1).

**Table 1:** Comparison of the descriptive characteristics between the training cohort and the validation cohort

Characteristics		Training	%	Validation	%	*P* value
Age at diagnosis (years)	N	3158		1053		0.947
		48.69±10.45		48.66±10.52		
BMI (kg/m^2^)	N	2476		805		0.256
		23.32±3.24		23.47±3.38		
Tumor location	N	2831		937		0.584
	UIQ	493	17.41	172	18.36	
	UOQ	1359	48	459	48.99	
	LIQ	168	5.93	60	6.4	
	LOQ	277	9.78	95	10.14	
	central	173	6.11	49	5.23	
	others*	361	12.75	102	10.89	
Clinical tumor size^1^	N	2668		898		0.592
	T1	783	29.35	277	30.85	
	T2	1572	58.92	524	58.35	
	T3	313	11.73	97	10.8	
Local invasion^2^	N	2745		918		0.842
	Yes	136	4.95	47	5.12	
	no	2609	95.05	871	94.88	
Pathological type	N	3001		1013		0.776
	DCIS-Mi	93	3.1	30	2.96	
	IDC	2585	86.14	873	86.18	
	ILC	105	3.5	30	2.96	
	others**	218	7.26	80	7.9	
Clinical lymph node status^3^	N	2803		907		0.080
	N0	1704	62.81	599	66.04	
	N1-N3	1099	37.19	308	33.96	
ER	N	2641		893		0.370
	Positive	1527	57.82	501	56.1	
	Negative	1114	42.18	392	43.9	
PR	N	2641		893		0.306
	Positive	1551	58.73	507	56.77	
	Negative	1090	41.27	386	43.23	
HR	N	2641		893		0.881
	Positive	1788	67.7	607	67.97	
	Negative	853	32.3	286	32.03	
HER-2 receptor status	N	2131		718		0.589
	Positive	556	26.09	180	25.07	
	Negative	1575	73.91	538	74.93	
Molecular subtype	N	2447		830		0.604
	LM	1788	73.07	607	73.13	
	HER2+	219	8.95	66	7.95	
	TN	440	17.98	157	18.92	
Multifocality^4^	N	2459		833		0.682
	Multifocal	84	3.42	26	3.12	
	Unifocal	2375	96.58	807	96.88	
ALN^5^	N	2926		974		0.644
	Positive	1426	48.74	483	49.59	
	Negative	1500	51.26	491	50.41	

^1^Clinical tumor size assessment by preoperative ultrasound

^2^Local invasion: invasion of skin or chest wall

^3^Clinical lymph node status assessment by preoperative palpation or imaging

^4^Multifocality: assessment by ultrasound or mammography

^5^ALN: examined postoperatively with H&E and IHC staining

*others: occult breast cancer or tumor cannot be touched in the breast

**others: tubular carcinoma, mucinous carcinoma, medullary carcinoma

Abbreviations: UIQ, upper inner quadrant; UOQ, upper outer quadrant; LIQ, lower inner quadrant; LOQ, lower outer quadrant; BMI, body mass index; HR, hormone receptor; ER, estrogen receptor; PR, progesterone receptor; DCIS-Mi, ductal carcinoma in situ with micro-invasion; IDC, invasive ductal carcinoma; ILC, invasive lobular carcinoma; LM, luminal-like; TN, triple-negative; HER-2, human epidermal growth factor receptor-2; ALN, axillary lymph node

### Univariate logistic regression analysis of ALN metastasis in the training cohort

Univariate logistic regression analysis was used to explore ALN metastasis-related variables (Table 2) and showed that age, tumor size, primary tumor quadrant, clinical nodal status, local invasion status, pathological type, ER status, and molecular subtypes were related to breast cancer ALN metastasis (*p* < 0.05).

**Table 2:** Univariate analysis for factors associated with axillary lymph node metastasis

Variables	Coefficient	SE	OR	95%CI Lower	95%CI Upper	*P* value
Age at diagnosis (years)	0.008	0.004	0.992	0.992	0.999	0.037
BMI (kg/m2)	0.011	0.013	1.101	0.985	1.037	0.399
Local invasion	0.834	0.212	2.303	1.519	3.493	0.000
ER	0.118	0.298	1.656	1.164	2.356	0.005
PR	0.002	0.081	1.002	0.856	1.174	0.976
HR	0.107	0.085	1.112	0.942	1.314	0.210
HER-2	0.201	0.212	1.223	0.981	1.824	0.066
Multifocality	0.202	0.228	1.224	0.783	1.914	0.376
Tumor location						
UIQ versus Central	-1.004	0.190	0.366	0.253	0.531	0.000
UOQ versus Central	-0.453	0.173	0.635	0.453	0.892	0.009
LIQ versus Central	-0.991	0.232	0.371	0.236	0.584	0.000
LOQ versus Central	-0.229	0.205	0.796	0.533	1.188	0.264
Others versus Central	-0.538	0.197	0.584	0.397	0.860	0.006
Molecular subtype						
LM versus TN	0.188	0.109	1.207	1.974	2.495	0.035
HER-2+ versus TN	0.121	0.169	1.129	0.811	1.571	0.473
Clinical lymph node status	1.436	0.088	4.203	3.534	4.997	0.000
Clinical tumor size						
T2 versus T1	0.360	0.090	1.433	1.201	1.711	0.000
T3 versus T1	1.136	0.154	3.115	2.303	4.213	0.000
Histological type						
IDC versus DCIS-Mi	2.219	0.376	9.194	4.403	19.197	0.000
ILC versus DCIS-Mi	1.940	0.423	6.961	3.039	15.942	0.000
Others versus DCIS-Mi	1.476	0.402	4.376	1.991	9.615	0.000

Abbreviations: UIQ, upper inner quadrant; UOQ, upper outer quadrant; LIQ, lower inner quadrant; LOQ, lower outer quadrant; BMI, body mass index; HR, hormone receptor; ER, estrogen receptor; PR, progesterone receptor; DCIS-Mi, ductal carcinoma in situ with micro-invasion; IDC, invasive ductal carcinoma; ILC, invasive lobular carcinoma; LM, luminal-like; TN, triple-negative; HER-2, human epidermal growth factor receptor-2; OR, odds ratio; CI, confidence interval; SE, standard error

### Processing of missing data and multivariate logistic regression analysis of ALN metastasis in the modeling group

Because of the longer duration of data collection, a large amount of data and collecting information, partial data were missing. We found no significant difference in the clinical and pathological features of patients with missing data between the two groups (*p* > 0.05; Table 3). Further, patients who did not undergo ALND or SLNB were excluded (*n* = 232 in the training cohort [7.35%] and *n* = 79 in the validation cohort [7.5%]). Considering that molecule subtype included ER status, the variable ER was excluded in multivariate regression analysis. Finally, 1869 and 642 patients with complete data on age, tumor size, primary tumor quadrant, clinical lymph nodes, local invasion status, pathological type, molecular subtypes were included in the training cohort and validation cohort, respectively (Figure 2). Multivariate analysis confirmed that age, tumor size, primary tumor quadrant, clinical nodal status, invasion of the chest wall and skin, pathological type, and molecular subtype were independent predictors of ALN metastasis (Table 4).

**Table 3:** Comparison of the clinical and pathological features of patients with missing data between the two groups

Variables	Training	Percentage (%)	Validation	Percentage (%)	*P* value
Age at diagnosis	0	0	0	0	-
Tumor location	327	10.35	116	11.02	0.545
Clinical tumor size	490	15.52	155	14.72	0.534
Local invasion	413	13.08	135	12.82	0.830
Pathological type	157	4.97	40	3.8	0.119
Clinical lymph node status	445	14.09	146	13.87	0.855
Molecular subtype	711	22.51	223	21.18	0.366

**Figure 1:**
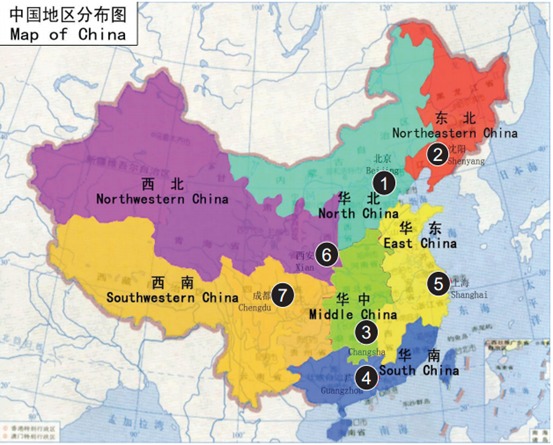
Geographic distribution of sites included in the study The numbers in the map represent the following: 1: Cancer Institute/Hospital, Chinese Academy of Medical Sciences, 2: Liaoning Cancer Hospital, 3: Second Xiangva Hospital, Central South University, 4: Guangdong Sun Yat-Sen University Cancer Center, 5: Zhejiang Cancer Hospital 6: First Affiliated Hospital of Xi’an Jiaotong University, 7: Sichuan Cancer Hospital [20].

**Figure 2:**
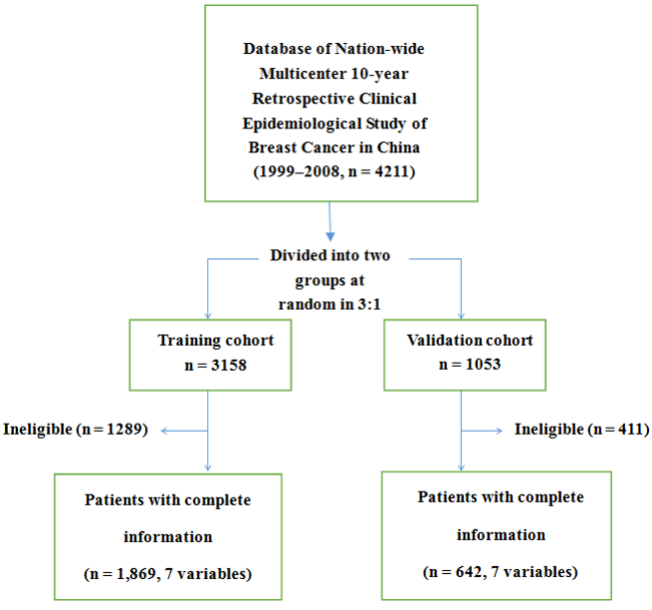
Patients with complete information in the training cohort and the validation cohort The 7 variables denote age at diagnosis, clinical tumor size, tumor location, clinical lymph node status, local invasion, pathological type, and molecular subtype.

**Table 4:** Multivariate logistic regression analysis for factors associated with axillary lymph node metastasis

Variables	Coefficient	SE	OR	95%CI Lower	95%CI Upper	*P* value
Age at diagnosis (years)	-0.014	0.005	0.986	0.976	0.995	0.004
Clinical tumor size						
T2 versus T1	0.204	0.112	1.226	0.985	1.528	0.069
T3 versus T1	0.663	0.210	1.940	1.286	2.927	0.002
Tumor location						
UIQ versus Central	-0.944	0.252	0.389	0.237	0.638	0.000
UOQ versus Central	-0.529	0.230	0.589	0.375	0.926	0.022
LIQ versus Central	-1.444	0.321	0.236	0.126	0.443	0.000
LOQ versus Central	-0.237	0.267	0.789	0.467	1.332	0.375
Others versus Central	-0.642	0.261	0.526	0.315	0.878	0.014
Local Invasion	0.768	0.314	2.156	1.166	3.986	0.014
Clinical lymph node status	1.235	0.109	3.440	2.777	4.261	0.000
Histological type						
IDC versus DCIS-Mi	2.944	0.624	18.998	5.595	64.509	0.000
ILC versus DCIS-Mi	2.884	0.674	17.887	4.778	66.964	0.000
Others versus DCIS-Mi	2.111	0.658	8.254	2.273	29.972	0.001
Molecular subtype						
LM versus TN	0.322	0.135	1.380	1.059	1.799	0.017
HER-2+ versus TN	0.141	0.210	1.152	0.764	1.737	0.500

Abbreviations: UIQ, upper inner quadrant; UOQ, upper outer quadrant; LIQ, lower inner quadrant; LOQ, lower outer quadrant; DCIS-Mi, ductal carcinoma in situ with micro-invasion; IDC, invasive ductal carcinoma; ILC, invasive lobular carcinoma; LM, luminal-like; TN, triple-negative; HER-2, human epidermal growth factor receptor-2; OR, odds ratio; CI, confidence interval; SE, standard error

### Establishment of a prediction model for ALN metastasis

**Figure 3:**
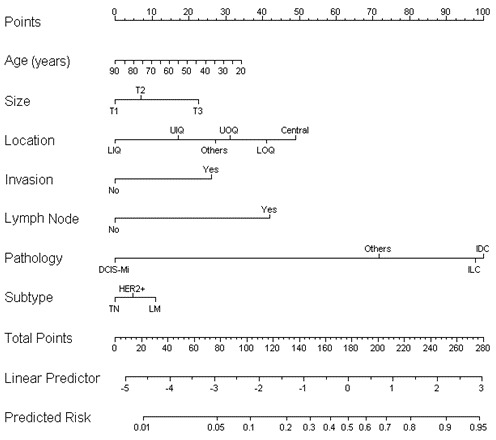
Nomogram for predicting the probability of axillary lymph node metastasis Age(years)- age at diagnosis in years; Size- clinical tumor size; Location- the location of tumor; Invasion- invasion of skin or chest wall; Lymph node- clinical lymph node status; Pathology- Pathological type; Subtype: molecular subtype There are a total of 11 rows in the nomogram. The behavioral variables are presented in rows 2 to 8, and points for each variable are correspond the scale in row 1. The points of the seven variables are added to the total points presented on the scale in row 9, which corresponds to the linear predictor and risk predictor of axillary lymph node metastasis in rows 10 and 11, respectively.

**Figure 4:**
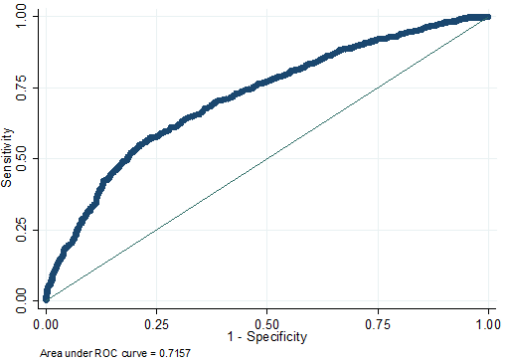
ROC curve of the predictive model for the training cohort (n = 1869) (ROC curve with an AUC value of 0.7157) ROC, receiver-operating characteristic ROC; AUC, area under the ROC curve.

According to the results of multivariate analysis, the following seven variables were included in the prediction model of ALN metastasis: age, tumor size, primary tumor quadrant, clinical nodal status, local invasion status, pathological type, and molecular subtypes. The weights of each variable in the model corresponded to different points (Figure 3). Points for the following factors were added to the total points, which corresponded to the linear predictors and risk predictors of ALN metastasis (Figure 3): size (T1, 0; T2, 7; T3, 23), location (LIQ, 0; UIQ, 17; UOQ, 31; LOQ, 41; central, 49; others, 27), invasion (no, 0; yes, 26), lymph node (no, 0; yes, 42), pathology (DCIS-Mi, 0; ILC, 98; IDC, 100; others, 72), subtype (TN, 0; HER2+, 5; LM, 11). According to the results of multivariate logistic regression analysis, the ALN metastasis risk of patients was expressed by the following equation:

ln(p/1−p) = -0.014 × a + 0.204 × b2 + 0.663 × b3 - 0.944 × c1 - 0.529 × c2 - 1.444 × c3 - 0.237 × c4 - 0.642 × c5 + 0.768 × d +1.235 × e + 2.944 × f1 + 2.884 × f2 + 2.111 × f3 + 0.322 × g1 + 0.141 × g2 - 2.483

where “p” represents the risk of ALN metastasis, “a” represents age at diagnosis, “b” represents tumor size (b2.T2; b3.T3), “c” represents tumor site (c1.UIQ; c2.UOQ; c3.LIQ; c4.LOQ; c5.others), “d” represents local invasion; “e” represents clinical lymph node status, “f” represents pathological type (f1.IDC; f2.ILC; f3.others), and “g” represents the molecular subtype (g1.LM; g2. HER2 +). This model was retrospectively utilized for patients in the training cohort (*n* = 1869), with an AUC value of 0.7157 (Figure 4), suggesting that it had a good predictive ability.

### Prospective applications of the prediction model of ALN metastasis

**Figure 5:**
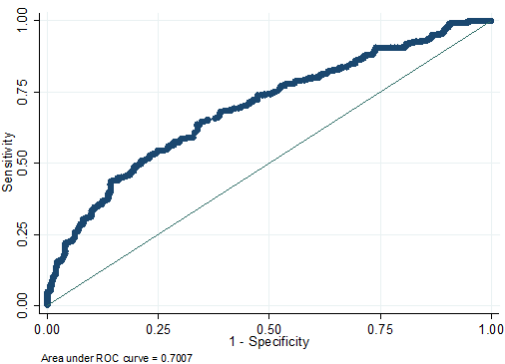
ROC curve of the predictive model for the validation cohort (n = 642) (ROC curve with an AUC value of 0.7007) ROC, receiver-operating characteristic ROC; AUC, area under the ROC curve.

**Figure 6:**
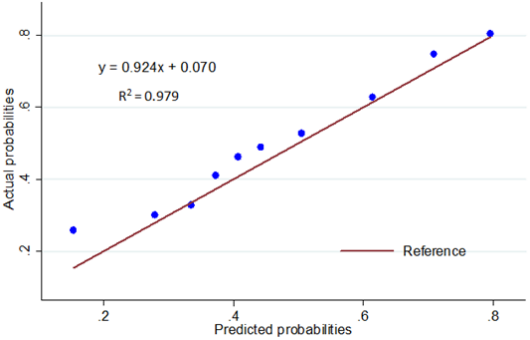
Calibration plot for the predictive model: The actual probability versus the predicted probability The reference line represents perfect equality of the predicted probability and the actual incidence of ALN metastasis.

This prediction model of ALN metastasis was prospectively used for patients in the validation cohort. It depicts the ROC curve, and the AUC value calculated was 0.7007 (Figure 5), indicating a good predictive ability. As seen in Figure 6, the curvilinear trend of predicted values and the real value was the same; there was no significant deviation, indicating that the predicted risk of ALN metastasis was consistent with the actual metastasis risk. The coefficient of determination represented the accuracy of model, and the R^2^ value of the model was 0.979, suggesting good calibration. On further evaluation of the clinical value of the model using cutoffs, we found that when the cutoff values of 14.03% and 20% were considered, the false-negative rates of model were 0 and 6.9%, respectively (Table 5).

**Table 5:** Accuracy of the developed model in low-risk predictive patients in the validation cohort

Predicted Risk (%)	No. of patients* (%)	Number of patients with ALN metastasis	Sensitivity (%)	Specificity (%)	Accuracy (%)	FNR (%)
<10.00	14 (2.18)	0	100	0	100	0
<14.03	21 (3.17)	0	99.56	4.86	100	0
<20.00	29 (4.52)	1	99.56	4.96	93.1	6.9
<22.01	49 (7.63)	2	98.45	8.06	89.8	10.02
<25.02	78 (12.15)	12	96.12	14.36	84.62	15.38
<31.27	104 (16.2)	19	92.12	25.00	81.37	18.27
<35.00	159 (24.77)	37	87.35	34.30	76.73	23.27
<40.00	244 (38.01)	68	76.58	51.55	72.13	27.87
<41.00	261 (40.65)	75	74.36	54.96	71.26	28.74
<41.50	265 (41.28)	77	73.03	56.51	70.94	29.06

A total of 642 patients had complete data in the validation group: 319 patients had actual positive axillary lymph node (49.69%) and 323 patients had actual negative axillary lymph node (50.31%)

*patients: the patients whose predicted risk is lower than the cutoff value

Abbreviations: FNR: false negative rate; ALN: axillary lymph nodes

## DISCUSSION

Breast cancer ALN status is a key factor in deciding the therapeutic options for patients and affects the prognosis of patients [3–5]. With more in-depth research on breast cancer, researchers consider ALND important for lymph node staging but has a small significance for treatment [28]. Therefore, if the ALN status of patients with breast cancer can be assessed in a noninvasive and accurate manner, clinicians can avoid surgical trauma and associated complications for low-risk patients with lymph node metastasis. Clinical research in medical centers in China and abroad has focused on the prediction of ALN metastasis status. In 2003, the MSKCC in the US established two models. The models were validated in many medical centers, but the results were widely inconsistent among different populations [14–19]. Therefore, to improve the accuracy of prediction, some clinicians used preoperative breast ultrasound, mammography photography, and breast magnetic resonance imaging (MRI) to predict the risk of ALN metastasis. However, the false-negative rate of prediction of the ALN status by ultrasound was 16.7-22.9%, After combining with mammography photography, breast MRI, and positron emission tomography/computed tomography (PET/CT), the false-negative rate reduced to 14-16.9% [29, 30]. However, thus far, there is no international consensus on the preoperative routine use of MRI [31]. In China, some clinicians used ultrasound in combination with clinical data of patients to build a prediction model of ALN metastasis and obtained an AUC value of 0.864, indicating a good predictive value [32]. However, the number of patients included in that model (*n* = 322 for the modeling group and *n* = 234 for the validation group) was relatively small, and the patients were from a single medical center; therefore, they only represented a small proportion of all patients in China. Thus, the development and application of this model in China were limited. In our current model, the seven breast cancer treatment centers included appropriately reflect the incidence, diagnosis, and treatment of breast cancer in women in China and represent the entire population of China [20].

In many risk predictions of cancer, the nomogram is considered an effective tool for quantitative assessment of risk factors to maximize the accuracy of prediction. It can reflect the contribution of predictive variables to the outcome visually and directly [33, 34]. In this study, we successfully established a nomogram model for prediction of breast cancer ALN metastasis that is suitable for Chinese people. Our results showed that the histopathological type played a crucial role in ALN metastasis, followed by tumor location, clinical lymph node status, age at diagnosis, invasion of the chest wall and skin, tumor size, and molecular subtype. Applying this model for patients in the training cohort and the validation cohort in this study, the performance of the nomogram in these two groups was similar (AUC = 0.7157 versus 0.7007), and the nomogram showed good predictive value in both. These results confirmed that our nomogram was useful in different population.

Several studies demonstrated that age of patients with breast cancer at diagnosis, BMI, tumor size, primary tumor quadrant, presence of multiple tumors, clinical lymph node status, local invasion status, pathological type, ER/PR, HER2 status, molecular subtypes, and other factors were related to ALN metastasis status [35–37]. Our results showed that the significance of these variables was similar to that of the results previously reported in the literature, including age at diagnosis, tumor size, clinical lymph node status, local invasion status, and pathological type.

In our study, tumor in the central region of the breast was more prone to ALN metastasis than that in other quadrants, which is consistent with the findings of other studies, showing abundant lymphatic drainage in the central region of the breast [38]. Some studies reported that the tumor in the UIQ of the breast was the most difficult tumor location of axillary metastasis [38–40]. However, we found that compared to the other locations, the risk of ALN metastasis in the LIQ was the lowest. We speculate that this difference between our study and previous studies is related to the differences in tumor heterogeneity, ethnic differences, lifestyle factors, and so on. The exact reason of this phenomenon remains unclear. Further, some studies showed that molecular subtypes had no predictive value for ALN status [41], while others showed that the triple-negative breast cancer patients had the lowest incidence of ALN metastasis and the HER-2 subtype had the highest incidence of ALN metastasis [42, 43]. Contradictory to these findings, we observed that luminal-like breast cancer patients were associated with higher probability of ALN metastasis. One possible explanation for this finding could be that Luminal like tumors had more lymphatic metastasis than triple-negative phenotype [32, 38, 44]. Nevertheless, these inconsistencies between our study and previous studies need to be investigated further.

To further assess the clinical application of the prediction model of ALN metastasis, we selected certain cutoff values for predicted risk for use in patients in the validation cohort. For patients with a metastasis risk below the cutoff value, we believed there was a low risk of ALN metastasis. Therefore, as per our model, they could be considered to be free from SLNB and ALND. According to the report of American Society of Clinical Oncology (ASCO), a false-negative rate of 0-29% was reported for SLNB, and the average false-negative rate was 8.4% [45]. In our model, the false-negative rate was only 6.9% when the cutoff value of 20%, which is less than the average false-negative rate of SLNB. Therefore, our nomogram should be acceptable in medical practice. SLNB may not be necessary when the predicted risk is less than 20%, especially for senile patients with other internal diseases and a lower surgical tolerance, who would have a low probability of ALN metastasis but might be more likely to suffer from postoperative complications [46, 47]. Thus, application of this model can reduce the surgical risk and postoperative complications in breast cancer patients with a low risk of ALN metastasis.

This study has several strengths that have been highlighted below: (1) To the best of our knowledge, this is the first nomogram prediction model of breast cancer ALN status that considered multi-center data and represents the entire Chinese population with breast cancer. (2) Our prediction model included data on seven variables, which can be obtained by conventional preoperative examination. This information will greatly improve the clinical application of the prediction model without additional examinations and costs. It has important implications for patients in developing countries and economically less-developed regions. (3) The AUC value obtained from the prospective data is 0.7007, suggesting a good predictive ability. Therefore, this model can help clinicians weigh the risks and benefits of SLNB before surgery in order to avoid unnecessary SLNB and ALND for patients.

Despite our important findings and strengths, our study had a few limitations that need to be addressed. First, the established model was based on clinical data of patients with breast cancer in multi-centers over a duration of 10 years. Owing to a long duration of data collection and differences in regional culture and educational and medical levels, there may be unavoidable biases introduced. For example, some data were missing in the study. Although 4211 patients were included in the analysis, only 2511 patients with complete data for variables were entered in the final model (1869 patients in the training cohort and 642 patients in the validation cohort). Second, in general, the predictive value of the model is considered good when the AUC value is 0.7-0.8 and very good when the AUC value is 0.8-0.9. When we prospectively used the model for patients in the validation cohort (*n* = 655), the obtained AUC value was 0.7007. The AUC value of our study is not perfect due to the large amount of data from multiple centers, it still needs more clinical central and a larger sample size to further evaluate and improve the predictive ability of model. In the future, we will validate the predictive ability of the model in larger clinical studies.

In conclusion, age of patients, tumor size, primary tumor quadrant, clinical nodal status, local invasion status, pathological type, and molecular subtypes were independent predictors of ALN metastasis. The nomogram model established in this study could provide an accurate and objective tool to predict the risk of breast cancer ALN metastasis by quantitative indicators. The developed model is easy to use and has a good predictive ability in the Chinese population. For low-risk patients with ALN metastasis, it can avoid the trauma and postoperative complications associated with axillary surgery, thereby improving the quality of life in patients.

## PATIENTS AND METHODS

### Study design

Data were obtained from the Nationwide Multicenter 10-year (1999-2008) Retrospective Clinical Epidemiological Study of Breast Cancer in China, led by Cancer Hospital/Institute, Chinese Academy of Medical Sciences (CICAMS) and jointly included seven Grade Three A hospitals nationwide.

### Selection of regions and hospitals

To ensure that the samples were representative of the total population with breast cancer in China, we selected seven geographic regions across China, including North, North-East, Central, South, East, North-West, and South-West regions (Figure 1). These regions encompassed most of the country and represented different breast cancer burdens. A representative Grade Three A hospital was selected from each region based on the criteria used in our previous study [20]. Briefly, the criteria are listed below: (1) the city where the hospital is located must be an important city in the region; (2) participant hospitals must be leading public cancer hospitals and regional referral centers providing pathology diagnosis, surgery, radiotherapy, medical oncology, and routine follow-up care for patients with breast cancer; and (3) the source of the patient must be able to cover the corresponding research area in order to represent the region.

### Data collection and quality control

Employees who uniformly received professional and systemic training in Beijing were responsible for recording patient's information. The patient information was collected by standard case report forms (CRF) designed by the CICAMS and included data on general information, risk factors, diagnostic imaging tests, therapy models, and pathologic characteristics. The reliability and validity of the CRF were assessed by a preceding pilot study. The data were transmitted to Cancer Hospital/Institute, Chinese Academy of Medical Sciences and verified by EpiData (http://www.epidata.dk/). Specific details of this process are described in our previous studies [20].

In 1999-2008, each hospital randomly selected a month every year, and data of at least 50 female breast cancer patients were collected in this month (January and February were excluded from the random selection to eliminate any confounding effects of China's largest annual holiday). If the number of patients included was less than 50 in the selected month, the patients from the immediately preceding month and the immediately following month were included until the total number of patients in that year reached 50. If, in the selected month, the number of patients exceeded 50, they were all included in the study. As such, a total of 4211 patients with breast cancer were included in the study.

### Patients and variables

The 4211 patients included in this study were randomly categorized into a training cohort or validation cohort in a 3:1 ratio. Relevant clinical and pathological features were grouped according to international practice. Age at diagnosis and body mass index (BMI; weight [kg]/square of height [m^2^]) were considered continuous variables. The following features were considered categorical variables: clinical tumor size assessment by ultrasound (categorized as T1 ≤ 2 cm, 2 cm < T2 ≤ 5 cm, T3 > 5 cm); primary tumor quadrant (categorized into upper inner quadrant [UIQ], upper outer quadrant [UOQ], lower inner quadrant [LIQ], lower outer quadrant [LOQ], central, and others [occult breast cancer or tumor cannot be touched in the breast]); preoperative ALN assessment by palpation or imaging (categorized into non-clinical metastasis [N0] and clinical metastasis [N1-N3]; the patients whose regional lymph nodes could not be assessed [Nx] were excluded); invasion of the chest wall and skin (categorized as invasion and non-invasion); multifocal tumors were categorized as multifocal and unifocal; pathological types were categorized as ductal carcinoma in situ with micro-invasion (DCIS-Mi), invasive ductal carcinoma (IDC), invasive lobular carcinoma (ILC), and other types of invasive carcinoma (tubular carcinoma, mucinous carcinoma, medullary carcinoma); expression of estrogen receptor (ER), progesterone receptor (PR), and human epidermal growth factor receptor-2 (HER-2) (categorized as positive and negative). The outcome variable—postoperative ALN status—was categorized as positive (presence of one or more ALN metastasis) and negative (no metastasis). Molecular subtypes were divided into three categories: luminal-like subtype (ER+ and/or PR+, any HER2 status), HER-2+ subtype (ER-, PR-, HER2+), and triple-negative (ER-, PR-, HER2-) [21, 22]. All patients included in the study were female patients with breast cancer who were diagnosed by histopathology and underwent successful SLNB and ALND.

### Pathologic processing

All nodes were examined postoperatively with serial section H&E staining. IHC staining was performed to determine whether micrometastasis (0.2-2 mm cancer foci) existed or not when no cancer cells were identified on H&E staining. ER, PR were considered positive if immunostaining was positive in more than 1% of tumour cells. HER-2 positivity was defined as a score of 3+ on IHC or amplification on FISH [23–25]. The histological subtype categorization was based on the 1981 and 2003 histological classification criteria of the World Health Organization [26]. Specific details are described in our previous studies [20, 27].

### Statistical analysis

The mean, SD, median were calculated to describe continuous variables, and a constituent ratio was used to describe categorical variables. *T*-test was used for the comparison of continuous variables, and Chi-square test or Fisher's exact test was used for comparison of categorical variables. Using the clinical and pathological data of the training cohort, univariate logistic regression analysis was performed to explore ALN metastasis-related variables. Subsequently, multivariate logistic regression analysis was used to determine the variables that were independent influence factors of ALN metastasis and establish the nomogram of the prediction model for breast cancer ALN metastasis. Receive-operating characteristic (ROC) curves, areas under the ROC curves (AUC), sensitivity, specificity were used to evaluate the predictive ability of model. The ROC curve was prepared by retrospectively using the data of the training cohort and calculating the AUC value. This prediction model was prospectively used for patients in the validation cohort by the depicting the ROC curve and re-evaluating the accuracy of the prediction through the AUC value. To test the accuracy and stability of the model, the decile of predicted values of metastasis risk was segmented based on the data of the validation cohort, and the average metastasis risk was calculated in each segment. The predictive value was taken as the abscissa, and the average actual metastasis risk was taken as the ordinate to draw the calibration curve. To further evaluate the clinical value of the model, we considered certain cutoff values for prediction risk in patients in the validation cohort and calculated the corresponding accuracy and false-negative rate of the cutoff values in order to assess the screening indicators of low-risk patients with ALN metastasis.

All statistical analyses were performed using SPSS (version 19.0, Chicago, IL, USA), Stata (version 11.0, College Station, TX), and R software (version 3.1.0, Institute for Statistics and Mathematics, Vienna, Austria). A two-tailed *p* value < 0.05 was considered statistically significant. In this study, all data are reported in aggregates.

### Ethics statement

The research was approved by Institutional Review Board of the Cancer Foundation of China. Because of the retrospective nature of the study, we were unable to contact all patients or their families, In addition, considering that it will not pose a risk to patients included in the study, informed consent was not obtained. All patient identifiers were removed, as per the approved procedures. De-identified data were maintained in a secure database, to which only research team members had access.
